# Transcriptome Analysis of Maize Leaf Systemic Symptom Infected by *Bipolaris zeicola*

**DOI:** 10.1371/journal.pone.0119858

**Published:** 2015-03-17

**Authors:** Ming Liu, Jian Gao, Fuqiang Yin, Guoshu Gong, Cheng Qin, Kunhao Ye, Min Zhang, Xiaofang Sun, You Zhou, Youju Zhang

**Affiliations:** 1 College of Agricultural Science, Sichuan Agricultural University, Chengdu, China; 2 School of Agricultural Sciences, Xichang College, Xichang, China; 3 Key Laboratory of Biology and Genetic Improvement of Maize in the Southwest Region, Ministry of Agriculture; Maize Research Institute, Sichuan Agricultural University, Wenjiang, Sichuan, China; Pennsylvania State University, UNITED STATES

## Abstract

*Bipolaris zeicola* is a fungal pathogen that causes Northern corn leaf spot (NCLS), which is a serious foliar disease in maize and one of the most significant pathogens affecting global food security. Here, we report a genome-wide transcriptional profile analysis using next-generation sequencing (NGS) of maize leaf development after inoculation with *B*. *zeicola*. We performed High-Throughput Digital Gene Expression analysis to identify differentially expressed genes (DEGs) in resistant inbred Mo17 lines after infection with *B*. *zeicola* at four successive disease development stages—CP (contact period), PP (penetration period), IP (incubation period), and DP (disease period); the expression of the genes was compared with those in a CK (mock-treatment) control. In addition, a sensitive maize line (Zheng58) was used for the comparisons with the Mo17. Among all tested genes, 466 differentially expressed genes were identified in all libraries, and Kyoto Encyclopedia of Genes and Genomes (KEGG) pathway analysis of these genes suggested that they are involved in many biological processes related to systemic symptom development, such as plant hormone signal transduction, starch and sucrose metabolism, phenylpropanoid biosynthesis and photosynthesis. Our systematic analysis provides comprehensive transcriptomic information regarding systemic symptom development in fungal-infected plants. This information will help in furthering our understanding of the detailed mechanisms of plant responses to fungal infection.

## Introduction

*Bipolaris zeicola* (G. L. Stout) Shoemaker (an anamorph of *Cochliobolus carbonum* R. R. Nelson) can cause Northern corn leaf spot (NCLS), a widespread foliar disease of corn (maize) and grasses in many regions of the world. NCLS favors regions that experience the appropriate amount of high rainfall, relative humidity, and temperature [1]; in particular, warm environments (68 to 90 degrees Fahrenheit) with high humidity are particularly conducive to NCLS [2]. NCLS is an important factor limiting the production of corn; the harm caused is not less than that caused by Northern corn leaf blight or Southern corn leaf blight in some hilly and mountainous areas. This is true especially in Sichuan Province, China, due to the lack of resistant cultivars and the temperate mountain climate, which favors the disease; however, this disease is not considered a serious problem for corn production in other countries.

Northern corn leaf spot (NCLS) can infect the leaf, ear, husk and sheath of corn, and major outbreaks can cause severe losses in yield and quality. NCLS begins as small, circular to oval, reddish brown to tan lesions. Over time, the lesions can become more tan to grayish tan in color and be surrounded by a lightly to darkly pigmented border. Five pathogenic races (races 0 through 4) of the fungus have been described [3]. Race 0 cannot infect maize but might cause leaf spot on grasses. Race 1, which produces a host-specific toxin (termed HC toxin), has become rare in the USA because modern maize hybrids are not sensitive to its toxin [3]. However, Race 1 is among the most destructive pathogens of maize and can kill susceptible maize plants at any stage of development [4], causing spotting of the leaf, sheath and the stalk and molding of the ear; this race has become more prevalent in China in recent years. Hm1, a widespread disease- resistance (DR) gene is lacking in the maize host [5]. Moreover, maize lines that are resistant to Race 1 are more tolerant of the HC toxin than susceptible lines [6]. In resistant host lines, Race 1 is contained at the infection site, in the same fashion that the HC-toxin-deficient Race 1 is contained in susceptible hosts. Race 2 is common in nearly all maize-growing areas but rarely causes significant damage [7]. Race 3 is considered a significant threat [8], particularly in Pennsylvania and North Carolina [9] and is more frequent in the Appalachian Mountains from Georgia to Pennsylvania in the United States; Race 3 has also been reported in China, Japan, Nigeria and Germany [10]. Race 4 can produce leaf spot on inbred lines with a B73 background.

Maize is a major international agricultural commodity and an important source of protein and energy for humans and livestock, as well as the many maize genome and transcriptome resources are available in recent years, such as the bioinformatics arm of the maize genome sequencing project (http://www.maizesequence.org/)[11], qTeller (http://qteller.com/qteller3/generate_figures.php) [12] and maize maize eFP Browser (http://bar.utoronto.ca/efp_maize/cgi-bin/efpWeb.cgi) [13]. The elucidation of the genome sequences and structures of diverse organisms has led to the development of various high-throughput genome and transcriptome analytical tools. Many transcriptome profiles in plant cells have been characterized under various conditions. Transcriptome changes in virus-infected plants, including *Arabidopsis* [14,15], *Nicotiana* [16,17], *Zea mays* [18] and *Vitis vinifera* [19], have been analyzed to identify the genes that constitute the expression networks underlying disease symptoms and viral propagation. Although the number of analyzed gene-expression profiles in maize under various stresses continues to increase, limited information is available for profiles associated with *B*. *zeicola* infection.

In the present study, based on the dynamic development of *B*. *zeicola* on maize leaves by scanning electron microscopy and determinate of superoxide dismutase (SOD), polyphenoloxidase (POD), polyphenoloxidase (PPO), catalase (CAT), and phenylalanine ammonia-lyase (PAL) in leaf responsive to *B*. *zeicola* by enzyme assay in the resistance maize line Mo17, compared with the sensitive maize line (Zheng58). We analyzed the responses of maize leaf (Mo17) to infection by *Bipolaris zeicola* at the transcriptome level using next-generation deep sequencing approaches. We investigated changes in gene expression between fungal-infected samples and mock-inoculated samples at various stages of symptom development after *B*. *zeicola* infection, including CP, PP, IP, and DP. The results indicate that RNA transport and purine metabolism were suppressed during pathogenesis and that metabolic pathways and plant hormone signal transduction were significantly enhanced during pathogen infection. Our study thus provides insights that might help to reveal the molecular mechanism of systemic symptom development in maize, which would further our understanding of plant-pathogen interactions.

## Materials and Methods

### Seed sterilization and experiment design

Seeds of the high-resistance maize inbred line “Mo17” and the highly sensitive maize inbred line “Zheng58” were treated with 7% hypochlorite solution for 30 min. The seeds were then washed three times with sterilized water and then sown in pots containing autoclaved soil. The resulting plants were grown in a growth chamber with a photoperiod of 14 h light/10 h dark at day/night temperatures of 25°C /23°C and a relative humidity of 75%. Control plants were maintained under the same conditions. The B12 strain of *Bipolaris zeicola* was used as the inoculum. First, the fungus was raised on potato dextrose agar (PDA) plates at 25°C under darkness for 5 d. Spores were washed with sterilized water, and the spore suspensions were then spread on PDA plates. The plates were incubated under the same conditions until they were covered with pathogen mycelia. Spores were collected by washing the cultures with distilled water containing one drop of Tween 80 per 100 mL, and the resulting suspensions were adjusted to a density of 1 × 10^4^ spores per mL using a hemocytometer. All maize plants were inoculated at the six- to eight-leaf stage by spraying a suspension of conidia (approximately 3–5 mL per plant) around the leaf whorl of each plant using a pressurized sprayer. Sterilized water was used instead of the suspension as a control. The plants were covered with plastic bags to ensure high humidity, and samples were collected from each of three maize plants; these three leaves were combined to represent one replicate. Three independent replicates were collected for each sample. Infected leaves were collected at 6, 12, 24, 36, 48, 72, 96, and 120 h. Control samples were harvested from water-treated leaves incubated under the same conditions. For observation of B. zeicola infection using scanning electron microscope, the inoculated samples were cut into 5-mm pieces and then glued onto a slide using a conductive adhesive and placed into an environmental scanning electron microscope (FEI Quanta450, Czech Republic). The samples were observed under a low vacuum pressure working environment (70 Pa).

### Enzyme activity assays and statistical analysis

To determination of CAT, SOD, PAL, POD, and PPO in leaves responding to Bipolaris zeicola infection, CAT activity was assayed by measuring the initial rate of H_2_O_2_ disappearance using the method of Beers & Sizer (1952) [20]. The catalase assay reaction mixture contained 0.05 mM sodium phosphate buffer (pH 7.0), 20 μl/mL enzyme extract and 1 mM H_2_O_2_. The decrease in H_2_O_2_ was followed by measuring the decrease in A240, and activity [U (mg protein) ^−1^] was calculated using a molar absorption coefficient of 40 mM^−1^ cm^−1^ for H_2_O_2_. SOD activity was monitored according to a published method [21]. The reaction mixture contained 50 mM sodium phosphate buffer (pH 7.8), 100 μM EDTA, 20 μl/mL enzyme extract and 10 mM pyrogallol. Enzyme activity [U (mg protein)^−1^] was determined by monitoring the reaction mixture for 120 s (at 60-second intervals) at 420 nm in a spectrophotometer. PAL activity was assayed using the method of Sadisivam and Manickam (1992)[22]. Leaf material (200 mg) was homogenized in 2 ml of 25 mm borate buffer, pH 8.8, containing 2 μl β-mercaptoethanol and a pinch of polyvinyl polypyrrolidone (PVP). The homogenate was filtered through cheesecloth and centrifuged at 12,000 × g for 10 min; the supernatant was then subjected to an enzyme activity assay using the method described by Sadisivam and Manickam (1992)[22]. One unit of enzyme was defined as an increase in absorbance of one unit per min. The activity of the enzyme was expressed as units per mg of soluble protein. Peroxidase (POD) activity was determined using the method of Upadhyaya et al. [23] in a 3.9 ml reaction mixture containing 50 mM phosphate buffer (pH 7.0), 28 μl guaiacol, 100 μl enzyme extract and 19 μl H_2_O_2_. The absorbance was monitored at 420 nm for at least 2 min at 30-second intervals; an absorbance change of 0.01 represented one unit of POD activity. Polyphenol oxidase activity (PPO) was assayed using 4-methylcatechol as substrate according to the method of Zauberman et al. [24]. One-half gram of fresh leaf was ground with 10 ml of 0.1 mol/l sodium phosphate buffer (pH 6.8) and 0.2 g of polyvinylpyrrolidone (PVP, insoluble). After centrifugation at 19,000 g for 20 min, the supernatant was collected and used as the crude enzyme extract. The assay reaction mixture included 1 ml of 0.1 mol/l sodium phosphate buffer (pH 6.8), 0.5 ml of 100 mmol/l 4-methylcatechol, and 0.5 ml enzyme solution. The increase in absorbance at 410 nm was recorded for 5 min at 25°C. One unit of enzyme activity was defined as an increase of 0.01 in the absorbance at 410 nm per min per mg protein. The protein content in the enzyme extracts was determined according to Bradford [25] using bovine serum albumin (BSA) as the standard. Treatments were replicated three times, and each replicate contained 5 pots (i.e., 15 pots per treatment). Data for all enzyme arrays were statistically analyzed using excel.

### Preparation of Digital Expression Libraries and Solexa sequencing

Based on the enzyme assay results and our microscope observations, we collected samples from infected leaves at selected time points from 0 h to 48 h as follows: CK (0 h) CP (12 h), PP (24 h), IP (36 h), and DP (48 h); the samples were pooled for RNA isolation and subsequent library construction. Comparable control leaves were treated identically and in parallel. The Digital Gene Expression Tag Profiling Kit was used to prepare sequence tags according to the manufacturer’s protocol. We used biotin oligo(dT) magnetic beads to purify 20 μg of total RNA and obtained 6 μg of mRNA. Then, double-stranded cDNA was introduced into a cDNA fragment, which was digested using NlaIII endonuclease and these binging fragments with the sequences of CATG site and adjacent polyA tail in 3’ end. After using magnetic bead precipitation to purify these 3’ cDNA fragments, Illumina adapter 1 (GEX adapter 1) and Illumina adapter 2 (GEX adapter 2) were added to the new 5’ end and the 3’ end of the cDNA tag, respectively. The junction between Illumina adapter 1 and the CATG site was recognized by MmeI, produced fragment of 17 bp tags with adaptor 1 by cutting at downstream CATG site. These cDNA fragments represented the tag library. These single-chain molecules were bound to the Illumina sequencing chip (flow cell) and prepared for Solexa sequencing after denaturation. Sequencing was performed at Beijing Genomic Institution (BGI). We enriched the samples for the desired fragments by PCR amplification using Phusion; the amplification employed 15 cycles, which were performed using primers that were complementary to the adapter sequences. PAGE gel electrophoresis (6% TBE) was used to purify the 85-base strips, which were then digested; the resulting single-chain molecules were then bound to a Solexa sequencing chip (flow cell). The samples were subjected to sequencing by synthesis using four color-labeled nucleotides. Image analysis and base-calling were performed using Illumina Pipeline, and cDNA sequence tags were revealed after purity filtering, which was implemented as follows: Tags passing initial quality tests were sorted and counted. Millions of raw reads were generated at each tunnel using a sequencing length of 35 bp (target tags plus the 3’adaptor), and a single tag was derived from a single transcript for each molecule in the library. The DGE-Seq raw data files have been deposited in NCBI’s Sequence Read Archive (SRA) and are accessible through SRA Series accession number SRA197274, all the supplement files were combined in S1 File.

### Analysis digital gene expression (DGE) tags and Identification of differently expressed genes

Clean tags were obtained by filtering the adaptor sequences, low-quality sequences (containing ambiguous bases) were removed, and then the resulting sequences were mapped to the reference genome and genes available at ftp://ftp.maizesequence.org/pub/maize/release-5b[11]. Only tags with a perfect match or one mismatch were considered further, and these were annotated based on the reference genes. The expression level of each gene was estimated based on the frequency of clean tags and was then normalized to TPM (the number of transcripts per million clean tags)[26]; this is a standard method that is extensively used in DGE analysis. The expression level of each gene was measured using the normalized number of matched clean tags; KOG functional classification, Gene Ontology (GO) and pathway annotation and enrichment analyses were based on the NCBI COG (http://www.ncbi.nlm.nih.gov/COG)[27], Gene Ontology Database (http://www.geneontology.org/) [28] and the KEGG pathway (http://www.genome.jp/kegg/)[29], respectively.

### Identification of genes that are differently expressed in each different library compared with CK library

The probability that one gene G is equally expressed in two samples is calculated using the following formula:

$$p(x|y)=\left(\frac{N2}{N1}\right)\frac{\left(x+y\right)!}{x!y!{\left(1+\frac{N2}{N1}\right)}^{\left(x+y+1\right)}}\begin{array}{c} C(y\leq ymin|x)=\overset{y\leq ymin}{\underset{y=0}{\sum }}p(y|x)\\ D(y\leq ymin|x)=\overset{\propto }{\underset{y\geq ymin}{\sum }}p(y|x) \end{array}$$

N1 and N2 denote the total number of clean tags in two compared libraries, x represented the clean tags that map to gene G in control [CK (0h)] library, y represented the clean tags that map to gene G in the differential treat libraries, including CP (12h), PP (24h), IP (36h), DP (48h) library respectively. The P value indicates the significance of prospect differences of transcript accumulation. A combination of FDR<0.001 and the absolute value of log2-Ratio> = 1 were used as the threshold to determine the significance of differences in gene expression.

### GO and pathway enrichment analysis of DEGs

We obtained the GO terms for each maize gene using Blast2GO (version 2.3.5) (http://www.blast2go.org/) based on the default parameters. Blast2GO was also used to implement a GO functional enrichment analysis of certain genes by performing Fisher's exact test with a robust FDR correction to obtain an adjusted p-value between certain test gene groups and the whole genome annotation.

### Validation of DGEs using Real-time PCR

To detect the expression patterns of candidate genes, the high-resistance maize inbred line Mo17 and the highly sensitive maize inbred line Zheng 58 were used in this study [30]. Total RNA was isolated from maize leaf exposed to *B*. *zeicola* for 12, 24, 36 and 48 h. Control samples were harvested from water-treated leaves incubated under the same conditions. To validate the DGEs obtained from Solexa sequencing, 15 DGEs were subjected to quantitative real-time PCR analysis using an ABI7500 instrument. Actin1 (GRMZM2G126010) was used as the endogenous control, and cDNA was synthesized using 1 μg of total RNA. The corresponding primers were designed using Primer5 software and are listed in Table A in S1 File. The amplification programs were programmed according to the standard ABI7500 system protocol: 95°C for 30 s; 95°C for 5 s, 60°C for 30 s, 40 cycles, and followed by a thermal denaturing step to generate melt curves, which were used to verify amplification specificity. All reactions were run in triplicate, including the non-template controls. The threshold cycles (Ct) of each tested gene were averaged for triplicate reactions, and the values were normalized according to the Ct of the control products of the Actin1 gene. The statistical analysis was performed using the 2^-ΔΔCT^ method.

## Results

### Calibration of maize leaf system development in response to *B*. *zeicola*

**Fig 1:**
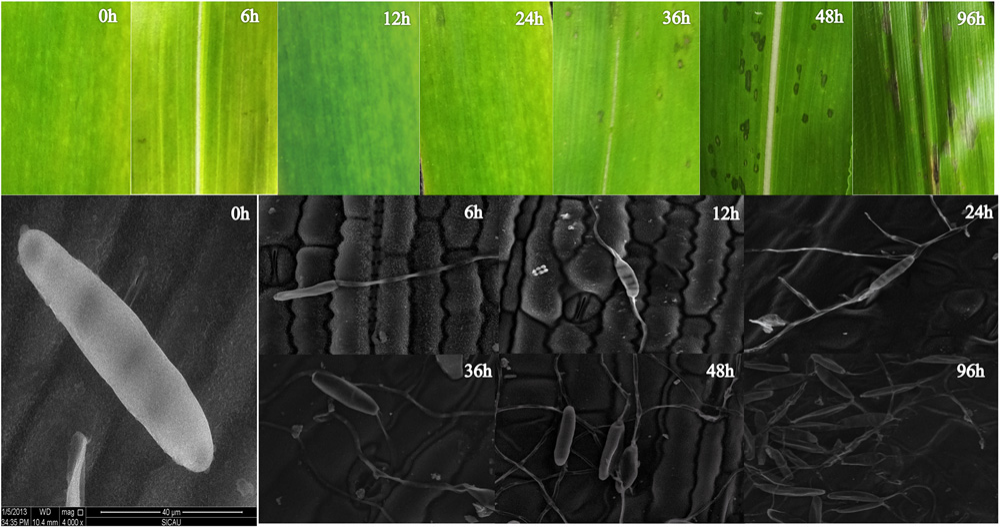
Defining the stages of maize leaf development in response to *B*. *zeicola* and observation of the successive stages of infection by *B*. *zeicola* under a scanning electron microscope. After inoculation, conidia of *B*. *zeicola* germinated, and one or two germ tubes were extruded from the poles of the conidia within 2~6 h. Appressorium-like structures were observed in contact with the maize leaf periderm after 12 h, at which time most of the structures had not yet penetrated into leaf surface cells; thus, this time point represents the contact phase (CP) of the disease development. At 24 h, numerous hyphae had differentiated from the germ tubes and were highly branched, and appressoria had directly penetrated the epidermal cell walls, in most cases by developing a constricted penetration peg. However, the fungus also entered through stomata and the intercellular space. This process was considered the penetration period (PP). At 36 h after inoculation, as observed during penetration, the fungus procured host nutrients and grew numerous mycelia on or underneath infected tissues. At the same time, some peridermal cells were collapsed, and a few infected tissues exhibited water-soaked spots, indicating that the infection had developed to the incubation period (IP). After 48h, more water-soaked spots appeared and more obvious symptoms were observed, and this was identified as the end of incubation period—disease periods (DP). The process of disease period was as follows: extensive mycelia colonized on leaf surface and the fungus appeared to have dissolved the cuticle as well as two suberized layers (arrowheads) that indicated mechanical pressure has occurred during host cell wall (HCW) penetration. At last, obvious necrosis appeared on the infected leaf at 96h after inoculation, Meanwhile, a great of new conidia reproduced on necrotic spots, it might be symptoms period (SP)

**Fig 2:**
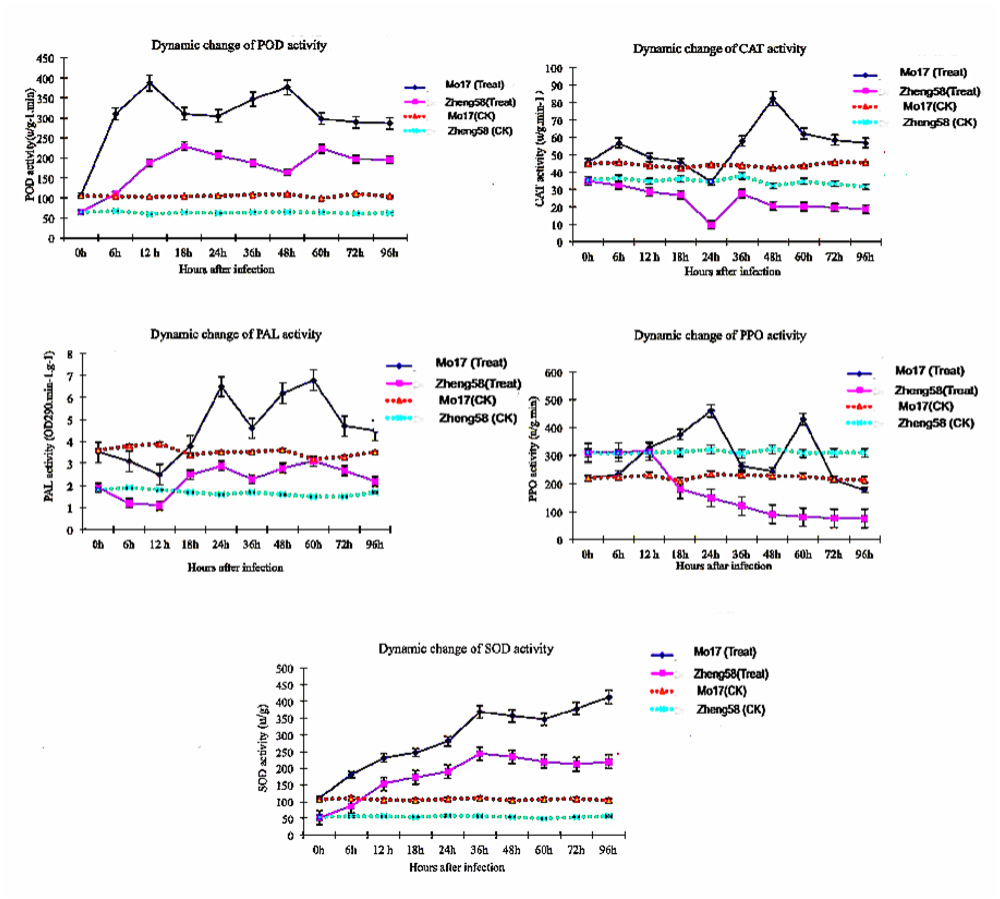
Determination of SOD, POD, PPO, CAT, and PAL enzyme activity in maize leaves responsive to *B*. *zeicola*.

To create inventories of gene expression at successive stages in leaf development in response to *B*. *zeicola*, the physiological characteristics of maize leaves were analyzed at successive stages. The dynamic development of *B*. *zeicola* on maize leaves was analyzed using scanning electron microscopy (Fig. 1), and the activities of SOD, POD, PPO, CAT, and PAL were also assayed on leaves infected with B. zeicola (Fig. 2). After inoculation, the conidia of *B*. *zeicola* germinated, and one or two germ tubes were extruded from the poles of the conidia within 2 to 6 h. Appressorium-like structures were observed in contact with the maize leaf periderm after 12 h, at which time most of the structures had not yet penetrated into leaf surface cells; thus, this time point represents the contact phase (CP) of the disease development. At 24 h, numerous hyphae had differentiated from the germ tubes and were highly branched, and appressoria had directly penetrated epidermal cell walls, in most cases by developing a constricted penetration peg. However, the fungus also entered through stomata and the intercellular space. This phase was considered the penetration period (PP). At 36 h after inoculation, as observed during penetration, the fungus procured host nutrients and grew numerous mycelia on or underneath infected tissues. At the same time, some peridermal cells were collapsed, and a few infected tissues exhibited water-soaked spots indicating that the infection had progressed to the incubation period (IP). After 48 h, more water-soaked spots appeared, and the symptoms became more obvious; this period was identified as the end of the incubation period (IP)-disease periods (DP). The process of disease period was as follows: extensive mycelia colonized on leaf surface and the fungus appeared to have dissolved the cuticle as well as two suberized layers (arrowheads) that indicated mechanical pressure has occurred during host cell wall (HCW) penetration. At last, obvious necrosis appeared on the infected leaf at 96h after inoculation, Meanwhile, a great of new conidia reproduced on necrotic spots, it might be symptoms period (SP) (Fig. 1).

In addition, we also measured the activity of isoenzymes of PPO, PLA, POD, SOD and CAT. The experimental results showed that the activity of these enzymes changed regularly, and the isozyme zymograms of the resistance-inbred line (Mo17) and the sensitive inbred line (Zheng 58) were different; Activities of all five enzymes in the un-inoculated Mo17 were higher than that in Zheng58. After fungal infection, the activation of SOD, PAL and POD increased remarkably, and the level of enzymes was greater in resistant Mo17 than in susceptible Zheng58. However, compared to control sample, the activities of CAT and PPO infected by fungi were much lower in infected zheng58.

### Characterization of the maize leaf transcriptome response to *B*. *zeicola*

**Fig 3:**
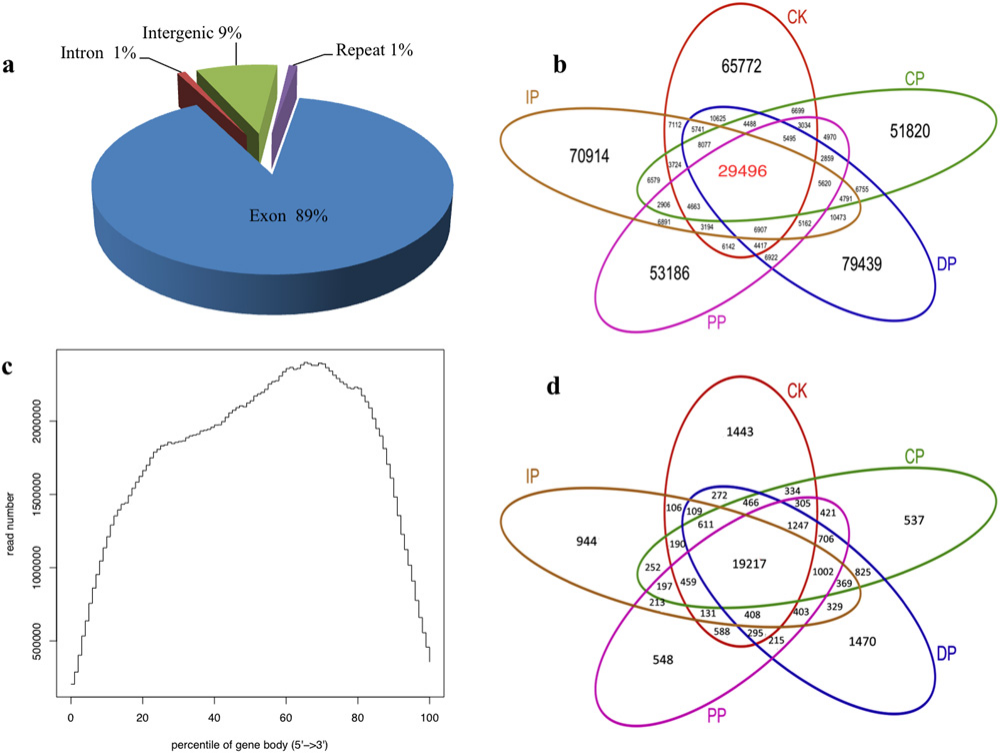
Digital Gene Expression (DGE) analysis of the Mo17 leaf transcriptome responding to *B*. *zeicola* infection. (a) Distribution of reads among the annotated genomic features of maize. (b) Shared and total reads among the phases of symptom development. (c) Distribution of reads among gene models and relative to transcript abundance. (d) Shared and unique reads among the phases of symptom development.

**Fig 4:**
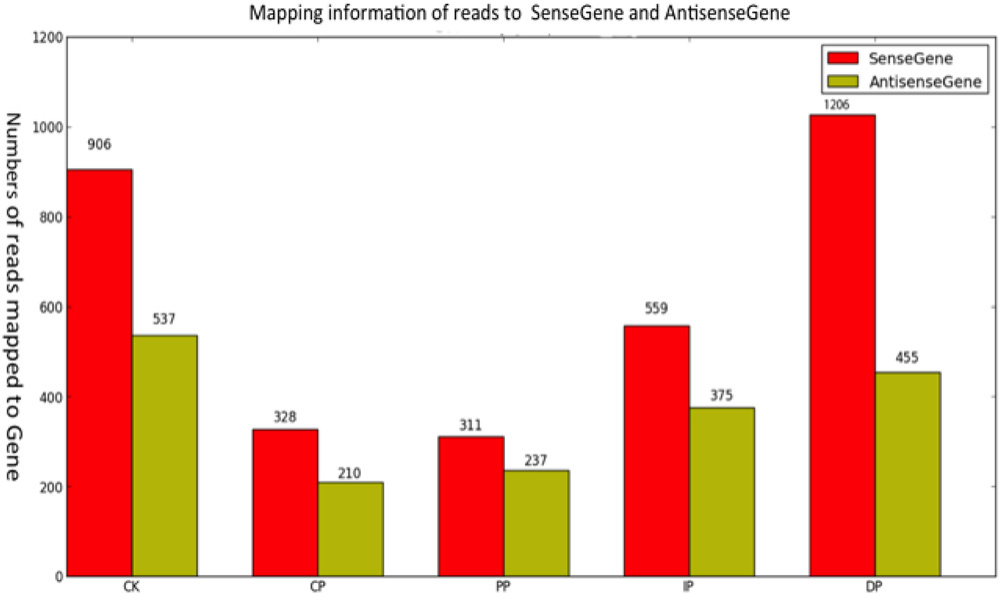
Mapping read information to sense and antisense genes at the five successive stages of symptom development.

In these experiments, we studied *B*. *zeicola*-inoculated leaves at 12 h (CP), 24 h (PP), 36 h (IP) and 48 h (DP), as well as mock-treated leaves (0 h) (CK). The development of leaves in response to *B*. *zeicola* was investigated in the resistant inbred line Mo17 and in mock-treated (0 h) plants using Digital Gene Expression technology. To profile the leaf transcriptome, we isolated mRNA from each of the five developmental stages, sheared it, and used it to prime cDNA synthesis (mRNA-seq). We constructed libraries and analyzed sequences using the Illumina platform. We generated approximately 47 million high-quality reads from the five developmental stages (Fig. 3b). The genic distribution of reads obtained using mRNA-seq showed that most (89%) of the reads mapped to protein-coding genes. The remaining reads were distributed among introns (1%), intergenic regions (9%) and repeat sequences (1%)(Fig. 3a, Table B in S1 File). To determine the distribution of reads throughout the body of the transcript, we visualized this trend by plotting the distribution of reads relative to cDNA ends (Fig. 3c). The mRNA-seq data reads were distributed uniformly, and a high percentage of reads mapped to genic regions. To estimate how many genes were expressed throughout leaf development in response to *B*. *zeicola*, we mapped the reads to sense genes and antisense genes and detected the following gene expression sequence from CP to DP: DP (20,687) >IP (20,161) >PP (19,765) >CP (19,754); mock-treatment (CK)(20,660). In total, 24,248 genes (74.2% of the annotated transcriptome for maize) are expressed during the development of maize leaf infection by B. *zeicola*; of these genes, 19,217 were expressed in all five sampled stages (Fig. 3d). Most reads mapped to sense genes rather than antisense genes (Fig. 4).

### Dynamic reprogramming of the maize leaf transcriptome at successive symptom stages

**Fig 5:**
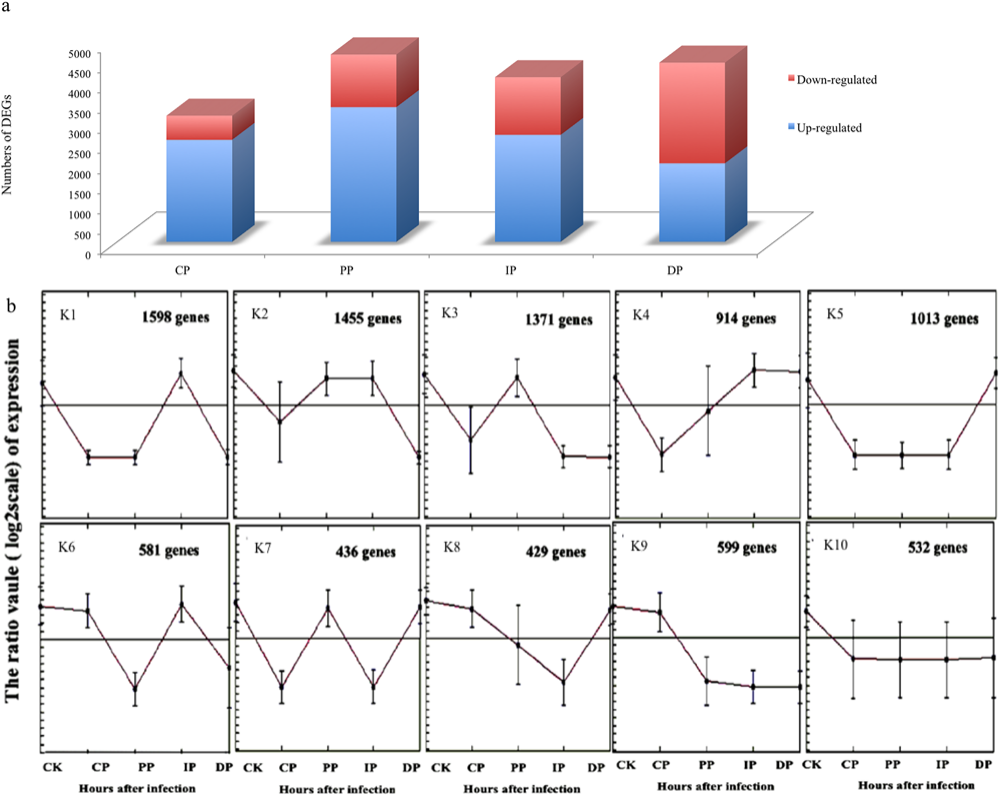
Numbers of differently expressed genes among the developmental stages of leaves infected with *B*. *zeicola* compared with mock-treatment (Fig. 5a); functional categories and genes grouped according to developmental dynamics using the K-Means clustering algorithm (Fig. 5b).

We identified that 16,276 out of 24,248 genes were differentially expressed among the developmental stages after inoculation with *B*. *zeicola* as compared with the mock-treatment control, representing 67% of the leaf transcriptome (Fig. 5a). We then assigned genes to functional categories and grouped the genes into 10 clusters according to developmental dynamics using the K-Means clustering algorithm (K1–K10; Fig. 5b). Five main clusters (K1–K5) accounted for approximately 72% of the differentially expressed genes in five successive stages (approximately 6,401 genes). Most of the functional annotations demonstrated the enrichment of particular gene expression clusters. For example, genes that encode enzymes for oxidoreductase activity and phosphatase activity are greatly enriched in cluster K1, representing genes that are expressed at the highest levels in the previous infection stage to combat the pathogen infection by reducing the toxic effect of oxides. Genes that show maximal expression during the transition infection time (clusters K2, K3 and K4) included those that are required for secondary cell wall biosynthesis and those that encode enzymes and transporters for starch metabolism, sucrose metabolism, and minor carbohydrates such as 5'-3' exonuclease activity, glucosyltransferase activity, ligase activity were also greatly enriched in this segment, suggesting that the leaf transcriptome undergoes substantial reprogramming as the leaf builds its cell wall to resist pathogen attack. Finally, the leaf gene expression is nearly exclusively committed to chlorophyll synthesis and plastid-to-nucleus signal transduction; genes that are predominantly expressed at DP stages (Cluster K5) encode enzymes for tetrapyrrole biosynthesis, such as tetrapyrrole activity, suggesting that the leaf rebuilds its photosynthetic machinery to combat pathogen infection.

### Changing inventories of transcription factors

**Fig 6:**
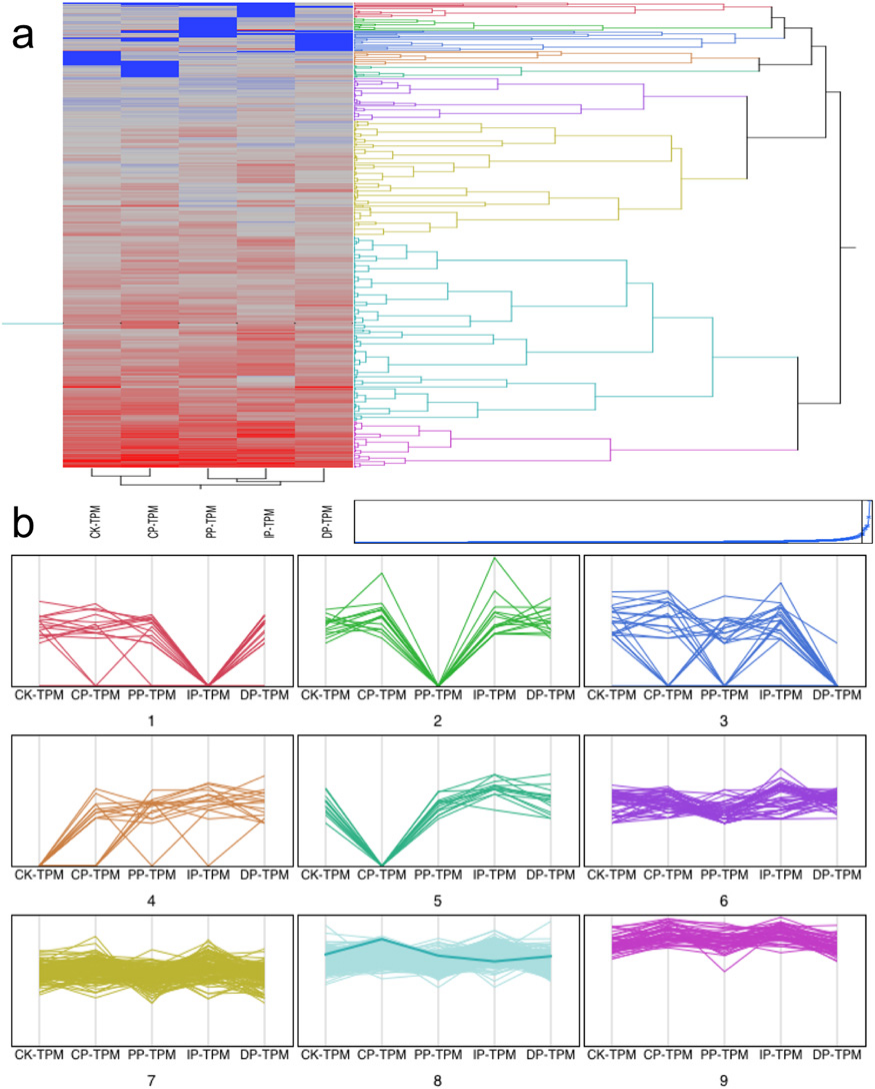
Cluster and parallel plot of transcription factors expressed at significantly different levels in the five successive stages of infection. (a) Clusters of transcription factors expressed at significantly different levels in the five successive stages of infection. (b) Parallel plot of transcription factors expressed at significantly different levels in the five successive stages of infection.

**Fig 7:**
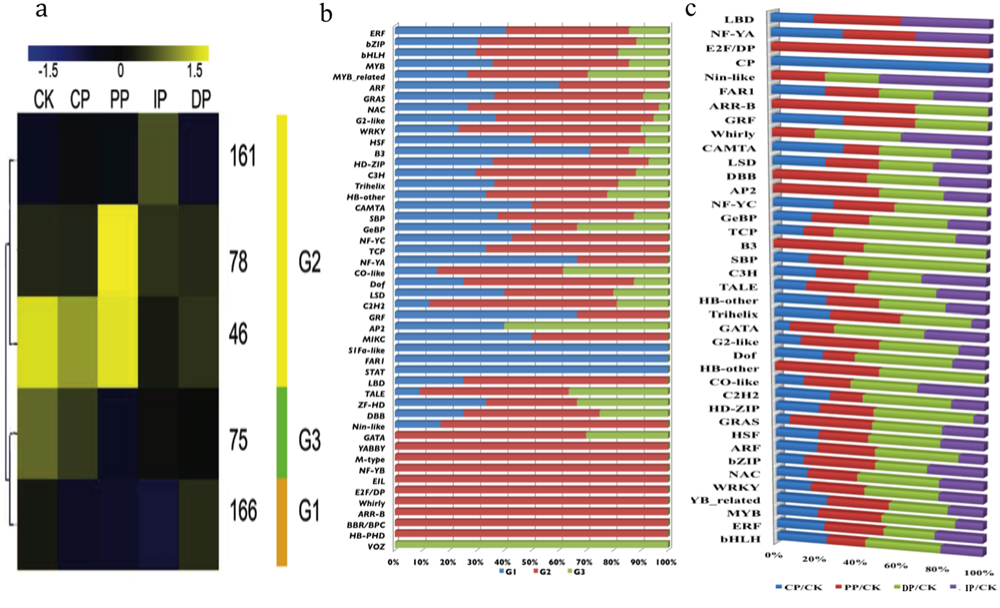
Dynamics of transcription factor accumulation profiles. (a) Dendrogram of transcription factors. Clustering of 527 transcription factors expressed at significantly different levels at the CK phase, during the transition from the CK to the DP phase, and the DP phase clustered into three lineages (G1, G2, and G3) using the self-organization tree algorithm (SOTA). (b) Distribution of transcription factor family proteins among G1, G2 and G3. (c) Distribution of transcription factor families that are expressed at different levels among the CK, CP, IP, PP and DP phases; of which, CP, IP, PP are the transition stage from CK to DP.

The dynamics of transcription factor accumulation during leaf development in response to *B*. *zeicola* infection were particularly well resolved in our DGE-seq data. We detected 1,048 transcription factors in leaf tissue; 526 were differentially expressed among the successive development stages and were categorized into 9 clusters based on hierarchical clustering and parallel plotting (Fig. 6a, 6b, Table C in S1 File). Additionally, we clustered transcriptional regulators into three distinct developmental clusters (G1–G3), which correspond well to the clustering of three major developmental stages based on the self-organization tree algorithm (SOTA); these stages are mock-treatment (CK), transition (from CK to DP) and DP. Most of these genes (54%) were expressed at the highest levels in stages from CP to IP (G2); only 31% of these genes were expressed at the highest levels in DP (G1). An additional 15% of these genes exhibited maximal expression in mock-treatment (G3) (Fig. 7a). Members of the Activating Protein (AP), CO-like and three amino acid loop extension (TALE) families of transcriptional regulators are highly expressed in the mock-treatment, where they most likely have a wide range of functions in leaf development, including regulating gene expression during early development and increasing the complexity of plant development and architecture. A number of transcription factors that regulate genes involved in sugar signaling and hormone signaling, including gibberellic acid (GA) and signaling factors (GATA family), were also highly expressed in the mock-treatment, and these factors partly control overlapping processes during plant development, such as greening, flowering time, and senescence. Several NAM, ATAF, and CUC (NAC), myeloblastosi (MYB), teosinte branched 1, cycloidea and PCF (TCP), Homeodomain leucine zipper (HD-ZIP) and Basic helix-loop-helix (bHLH) transcription factors accumulated to their highest levels during the transition from the CP to the DP infection stage, the stages during which secondary cell walls are being established and when light-mediated developmental progression is perhaps strongest. Transcriptional regulators including Golden 2 (G2-like) and DNA-binding with one finger (DOF) transcription factors, which have been shown to regulate photosynthetic gene expression, were also preferentially expressed during the CP to DP transition. Interestingly, many transcription factors accumulated only during the transition stage from CK to DP, such as YABBY(YAB), M-type, Nuclear transcription factor Y (NF-YB), ETHYLENE INSENSITIVE like (EIL), E2F/DP, whirly, APR-B, BBR/BPC(BASIC PENTACYSTEINE) and HB-PHD (Homeobox-plant homeodomain). ARF (auxin response factor), B3, NF-CA, and Growth related factor (GRF) were enriched during the DP stage (Fig. 7b, 7c). These transcription factors are putatively involved in plant hormone signal transduction. Far-red-impaired response (FAR1), Signal Transducer and Activator of Transcription (STAT) and MCM1, AGAMOUS, DEFICIENS and SRF (MADS), intervening keratin-like and C-terminal (MIKC) are only enriched during the DP stage; these factors regulate many aspects of growth, survival and differentiation in cells, including the signal transduction pathway used for light-regulated development.

### Important KEGG pathways and GO annotation of genes that are differentially expressed in response to *B*. *zeicola*

**Fig 8:**
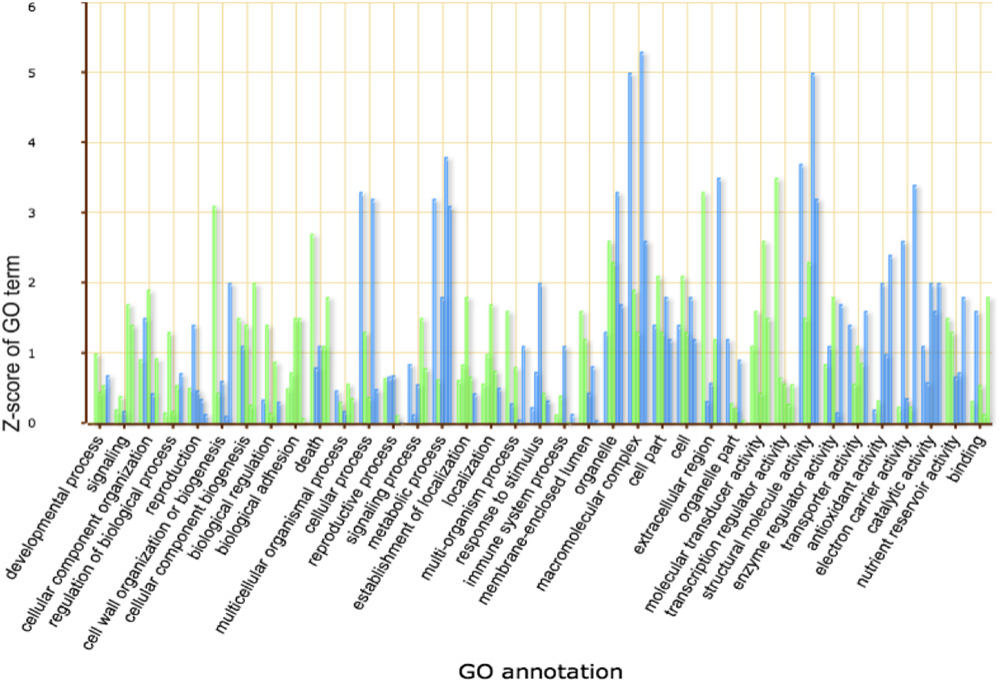
GO annotation of DGEs in leaf development in response to *B*. *zeicola* infection. The Y-axis represents the percentage of targeted genes mapped by the GO term and represents the abundance of the GO term. The X-axis expresses the definition of GO terms.

Interestingly, the KEGG pathway analysis showed that seven common KEGG pathways were significantly enriched in all four of the infection stages compared with the mock-treatment. Five of these pathways were up-regulated in plants showing increasing symptoms of *B*. *Zeicola* infection with time; these pathways were biosynthesis of secondary metabolites, plant hormone signal transduction, starch and sucrose metabolism, phenylpropanoid biosynthesis, and photosynthesis. Most of the significantly enriched KEGG pathways were related to metabolism, and most of these metabolic pathways were enhanced by *B*. *zeicola* infection; in contrast, pathways related to purine metabolism were inhibited at all four stages of symptom development. The important KEGG pathways influenced by *B*. *zeicola* infection are summarized in the tables (Table 1, Table D in S1 File). GO annotation of the differentially expressed genes showed that most of the enriched genes were related to the immune system, signaling and metabolism and that they were involved in antioxidant activity and transporter activity. However, reproduction and transcriptional regulation were inhibited at all four symptom development stages (Fig. 8).

**Table 1:** The 15 most enriched KEGG pathways during successive symptom phases and important KEGG pathways that are influenced by *B*. *zeicola* infection (there important KEGG pathways increased as the maize leaves developed in response to *B*. *zeicola* compared with mock-treatment (CK).

Pathway	CP/CK	PP/CK	IP/CK	DP/CK
Biosynthesis of secondary metabolites	131(8.89%)	232 (11.86%)	178 (12.07%)	147(13.36%)
Metabolic pathways	281(19.08%)	455 (23.26%)	343 (23.25%)	271(24.64%)
Plant hormone signal transduction	61 (4.14%)	86 (4.4%)	76 (5.15%)	69 (6.27%)
Plant-pathogen interaction	80 (5.43%)	85 (4.35%)	77 (5.22%)	43 (3.91%)
Starch and sucrose metabolism	25 (1.7%)	44 (2.25%)	32 (2.17%)	37 (3.36%)
Ubiquitin-mediated proteolysis	33 (2.24%)	35 (1.79%)	33 (2.24%)	33 (3%)
Protein processing in the endoplasmic reticulum	66 (4.48%)	57 (2.91%)	57 (3.86%)	44 (4%)
Circadian rhythm—plant	32 (2.17%)	40 (2.04%)	44 (2.98%)	22 (2%)
Spliceosome	70 (4.75%)	69 (3.53%)	45 (3.05%)	36 (3.27%)
RNA transport	37 (2.51%)	46 (2.35%)	31 (2.1%)	21 (1.91%)
Purine metabolism	37 (2.51%)	44 (2.25%)	32 (2.17%)	22 (2%)
Phenylpropanoid biosynthesis	10 (0.68%)	30 (1.53%)	25 (1.69%)	25 (2.27%)
Photosynthesis	7 (0.48%)	19 (1.29%)	32 (1.64%)	26 (2.36%)
Carbon fixation in photosynthetic organisms	14 (0.95%)	29 (1.48%)	17 (1.15%)	27 (2.45%)
Ribosome	60 (4.07%)	60 (3.07%)	16 (1.08%)	27 (2.45%)

### Comparison of DGE Tag Data with qRT-PCR Expression Patterns

To validate our DGE data, 15 unigenes with annotations were selected for qRT-PCR analysis (Table 2). The resulting qRT-PCR data for these genes were consistent with the DGE results. For example, the qRT-PCR and DGE analyses both showed that genes encoding pathogenesis-related protein 10 (PR10), ARF (auxin response factor), chitinase chem5 (chn1) and Antifreeze protein were expressed at significantly higher levels in *B*. *zeicola*-infected maize leaves than in uninfected leaves. Similarly, four pathogen-association genes, which were enriched in four *B*. *zeicola* infection stages, were found using DGE analysis and verified using qRT-PCR analysis; however, these genes (nudix hydrolase 13, nudix hydrolase 13, cytochrome b-c1 complex, and F-box protein) were expressed at lower levels compared with mock-treated samples. We further analyzed the expression of other seven pathogen-association genes that were increased at all four stages of infection by *B*. *zeicola* infection using qRT-PCR. These seven genes were among those that were neither significantly up-regulated nor significantly down-regulated during *B*. *zeicola* infection in the DGE dataset compared with mock-treatment, and these findings were consistent with the expression patterns obtained using qRT-PCR (Table 2).

**Table 2:** Verification of DGE-seq results by qRT-PCR.

			Development Stages responsive to *Bipolaris zeicola*				
Gene ID	Gene description	Material	CK	CP	PP	IP	DP
GRMZM2G324999	WRKY69	Zheng58(qRT-PCR)	0.858	0.467	3.324	4.716	5.224
		Mo17(qRT-PCR)	0.745	0.522	4.847	4.992	6.954
		Mo17(DGE)	1.3	0.01	1.46	4.72	5
GRMZM2G057116	WRKY DNA-binding protein	Zheng58(qRT-PCR)	3.571	2.863	20.37	21.743	45.442
		Mo17(qRT-PCR)	3.379	2.451	23.013	30.412	60.787
		Mo17(DGE)	3.7	0.66	10.19	10.68	13.7
GRMZM2G060109	bZIP	Zheng58(qRT-PCR)	7.239	1.681	2.202	7.632	8.171
		Mo17(qRT-PCR)	7.323	1.858	4.251	8.717	30.09
		Mo17(DGE)	6.31	1.98	3.54	5.55	15.87
GRMZM2G317584	EIN3	Zheng58(qRT-PCR)	11.139	10.749	12.872	20.932	25.124
		Mo17(qRT-PCR)	12.856	10.894	13.031	23.124	30.911
		Mo17(DGE)	15	10.98	15.81	21.31	28.31
GRMZM2G175480	bHLH	Zheng58(qRT-PCR)	2.202	1.681	7.323	7.632	8.717
		Mo17(qRT-PCR)	2.239	1.858	8.171	8.717	30.09
		Mo17(DGE)	0.01	0.01	1.25	1.85	27.18
GRMZM2G020150	ERF	Zheng58(qRT-PCR)	22.333	1.735	11.038	27.791	33.114
		Mo17(qRT-PCR)	22.282	2.053	10.483	26.002	40.254
		Mo17(DGE)	25.01	5.27	10.4	25.06	35.66
GRMZM2G042278	ARF	Zheng58(qRT-PCR)	2.306	3.418	15.514	39.548	54.554
		Mo17(qRT-PCR)	2.351	2.587	15.445	35.861	46.406
		Mo17(DGE)	5.44	5.71	13.31	32.86	36.75
GRMZM2G035103	C2H2	Zheng58(qRT-PCR)	30.269	1.713	11.149	30.269	44.726
		Mo17(qRT-PCR)	30.336	2.217	12.419	35.317	70.294
		Mo17(DGE)	29.57	4.61	13.52	24.85	61.53
GRMZM2G043275	nudix hydrolase 13	Zheng58(qRT-PCR)	5.65	1.156	2.261	4.022	5.044
		Mo17(qRT-PCR)	6.106	1.848	2.167	4.223	5.173
		Mo17(DGE)	18.7	1.76	2.7	4.31	5.87
GRMZM2G112524	pathogenesis-related protein 10	Zheng58(qRT-PCR)	1.008	7.376	7.747	18.286	28.032
		Mo17(qRT-PCR)	1.114	7.101	8.088	22.179	41.761
		Mo17(DGE)	1.3	7.03	8.11	15.82	21.53
GRMZM2G453805	PRm 3	Zheng58(qRT-PCR)	1.196	4.027	8.478	14.483	24.197
		Mo17(qRT-PCR)	1.622	4.172	9.481	16.183	32.488
		Mo17(DGE)	2.475	5.021	9.043	12.585	19.856
GRMZM2G542272	Antifreeze protein	Zheng58(qRT-PCR)	8.088	24.692	33.114	35.861	52.554
		Mo17(qRT-PCR)	9.431	29.039	30.046	34.068	60.09
		Mo17(DGE)	9.35	32.72	42.84	48.47	94.37
GRMZM2G479906	ABC transporter protein	Zheng58(qRT-PCR)	28.032	1.114	1.376	8.747	18.286
		Mo17(qRT-PCR)	31.761	1.008	3.481	7.747	18.088
		Mo17(DGE)	25.23	1.32	3.95	4.72	10
GRMZM2G023194	Cytochrome b-c1 complex	Zheng58 (qRT-PCR)	30.09	2.202	7.632	7.323	13.031
		Mo17(qRT-PCR)	34.068	7.239	8.717	8.171	14.911
		Mo17(DGE)	31.1	5.27	7.07	7.19	14.79
GRMZM2G154864	F-box protein	Zheng58(qRT-PCR)	15.022	5.908	6.938	8.945	14.966
		Mo17(qRT-PCR)	15.416	5.853	6.19	8.56	15.068
		Mo17(DGE)	36.32	5.27	6.24	8.22	12.83

## Discussion

Northern corn leaf spot (NCLS), which is caused by the fungus *B*. *zeicola* (Nisikado) Shoemaker (synonym = *Bipolaris zeicola* Nisikado and Miyake), is a serious foliar disease of maize that is distributed widely in maize-producing areas throughout the world. In China, the disease was first identified in 1972, and an outbreak in 1998 caused yield losses of between 42% and 53% of all corn products [31]. Currently, no known genes have been reported to confer complete immunity to this disease; thus, maize breeders rely on polygenic, quantitative resistance to NCLS to create resistant strains [32]. Several genes underlying QTLs for plant disease resistance have been cloned, representing a wide range of gene functions [33]. Although substantial progress has been made in elucidating the genetic basis of plant disease immunity conditioned by 'R genes' [34–36], little is known about the genes underlying systemic symptom responses to NCLS.

In this study, we used next-generation sequencing approaches to investigate the gene expression changes associated with the characteristic development of *B*. *zeicola* infection in maize. We mapped in detail the transcriptional changes that occur during leaf development in response to *B*. *zeicola* infection. We examined the dynamic development of *B*. *zeicola* on maize leaves using scanning electron microscopy and determined the activities of SOD, POD, PPO, CAT, and PAL in leaves in response to *B*. *zeicola* infection. We broadly subdivided the infection process into five successive symptom stages (CK, CP, PP, IP, and DP). Based on this grouping, we detected the expression of 24,248 genes or 74.2% of the annotated maize transcriptome. KEGG pathway analysis showed that 24,248 of the unigenes could be grouped into 127 known pathways. The ‘metabolic pathways’ group contained the largest number of genes (4,437 unigenes); the second largest group, ‘biosynthesis of secondary metabolites’, included 2,425 annotated unigenes; and the third largest group, ‘plant hormone signal transduction’, included 1,315 annotated unigenes. Based on the unigene reference dataset, our DGE analysis will provide further insights into the molecular mechanisms underlying systemic symptom development in maize leaves after *B*. *zeicola* infection. Analyses of differentially expressed genes (DEGs) and significantly enriched KEGG pathways for the different symptom stages indicated that several biological processes are influenced by pathogen infection. The process that is most affected is metabolism, and most of the annotated DEGs were correlated to metabolism and were enriched in KEGG pathways. Other biological processes that were significantly affected by *B*. *zeicola* infection during the development of symptoms included photosynthesis, starch and sucrose metabolism and plant-pathogen interaction.

In previous studies, it was reported that genes related to photosynthesis were suppressed by pathogen infection [37]. In our study, we observed that many genes in pathways related to photosynthesis, such as photosynthesis and carbon fixation in photosynthetic organisms, were up-regulated during the progression of pathogenesis (from CP to DP). Our results suggest that these pathways, especially those related to photosynthesis, might be directly responsible for maize leaf development during the response to pathogen attack. In addition, scanning electron microscopy analysis indicated that *B*. *zeicola* development might also be correlated to the development of symptoms in *B*. *zeicola*-infected maize leaves, and enzyme assays showed that SOD, PAL and POD activities are related to varietal disease resistance.

Many genes encoding the ‘plant-pathogen interaction’ pathway were up-regulated during symptom development; the enzymes encoded by these genes are involved in defense-related gene induction and innate immunity, such as those that activate genes coding for the WRKY transcription factor, pathogenesis-related protein 10, and PRm 3(ChitinaseChem 5), as reported previously [17,38,39]. Our results showed that genes encoding WRKY69 and WRKY DNA-binding protein were up-regulated at the PP, IP, and DP stages but were down-regulated at the CP stage compared with mock-treatment; PR10 and PRm 3 were up-regulated at the CP, PP, IP, and DP stages compared with CK. These genes were increasingly up-regulated during symptom development from CP to DP. WRKY proteins belong to a transcription factor family that exhibits a special structure in plants: one or two domains containing the sequence WRKYGQK [40], and these domains typically bind a cis-element termed a W box. A wide range of pathogens and defense hormones are activated by WRKY proteins [40]. In addition, several studies have described the role of transcription factors that contain a basic leucine zipper domain (bZIP) [41]. The family of transcription factors containing a bZIP domain is among the largest families of transcription factors in plants; as regulated family genes, bZIP family members regulate most of the genes involved in various processes, such as abiotic stress response, seed maturation, flower development and pathogen defense [42].

Moreover, many genes in the ‘plant hormone signal transduction’ pathway were up-regulated during symptom development. A negative effect of auxin signaling on plant resistance to biotrophic pathogens has recently been described [43]. Auxin regulates many processes during plant development through direct interaction with TIR1-like F-box receptor proteins [44], which, when bound to SCFTIR1, leads to the enhanced removal of members of the AUX/IAA family of transcriptional factor (TF) repressors through the SCF (Skp1–Cullin–F-box) E3-ubiquitin ligase proteasome pathway[45,46]. Thus, the degradation of AUX/IAA proteins allows the activation of Auxin Response Factors (ARFs) and the expression of auxin-responsive genes [47]. Moreover, an increasing body of evidence indicates that some plant pathogens either produce auxin themselves or increase plant auxin biosynthesis upon infection to manipulate host developmental processes.

bHLH factors function as transcription repressors by antagonizing transcription activators through binding to their target sequences; these factors include MYC2 and the WD-repeat/bHLH/MYB complex. The coordinated regulation of JA responses by transcription activators and repressors would benefit plants by allowing fine regulation of defense and development, allowing the plants to adapt to their frequently changing environment. Our results further revealed that several transcription factors, such as ARF (auxin response factor), F-box and bHLH transcription factors, accumulated to their maximum levels during the transition from the CP to the DP infection stage and were enriched at the DP stage. Furthermore, the plant hormone ethylene regulates a variety of stress responses and developmental adaptations in plants. This gas is well known to participate in a wide range of physiological processes, such as fruit ripening, senescence, abscission, germination, cell elongation, sex determination, pathogen defense response, wounding, nodulation, and cell fate determination [48,49]. Control of these processes by ethylene involves the complex regulation of ethylene biosynthesis and the ability of cells to perceive and respond to the hormone in an appropriate manner. Understanding the molecular events that lead to this diversity of plant responses is essential to elucidate how this gas modulates such functions. C2H2-type zinc finger proteins play crucial roles in many metabolic pathways and in the stress response and defense activation in plants. Recent studies have demonstrated the importance of C2H2-type zinc finger proteins due to their putative role in repressing the expression of genes that encode proteins that are involved in the defense and stress response of plants. Most of these defense and stress response proteins are thought to acquire their repression activity via their ethylene-responsive element-binding factor (ERF)-associated amphiphilic repression (EAR) domain (described below). Recent studies of the C1 C2H2-type zinc finger family have suggested that these proteins play key roles in several developmental pathways and in the defense and stress response pathways of Arabidopsis. EIN3 proteins comprise a family of novel sequence-specific DNA-binding proteins that regulate gene expression by binding directly to a primary ethylene response element (PERE); EIN3 is necessary and sufficient for ERF1 expression, and EIN3-overexpression can lead to the constitutive expression of ERF1 in transgenic plants, which in turn results in the activation of a variety of ethylene response genes and phenotypes. It has also been demonstrated that ERF1 can act downstream of EIN3 and all other components of the ethylene-signaling pathway. Surprisingly, we detected C2H2-type zinc finger, ERF and EIN3 in our DGE-seq data, and these data were validated using qRT-PCR; genes for these proteins increased during the infection stages and were enriched at the DP stage.

ABC transporters constitute one of the largest protein families found in all living organisms; these transporters are driven by ATP hydrolysis and can act as exporters as well as importers. Originally identified as transporters that are involved in detoxification processes, these transporters might be required for organ growth, plant nutrition, plant development, plant response to abiotic stress, pathogen resistance and the interaction of plants with their environment. We detected and validated ABC transporter protein expression in our study. Characterization of TaAbc1 expression revealed that the expression of this gene was tissue-specific and could be up-regulated by biotic agents (e.g., stripe rust pathogen) and/or by abiotic stresses, such as wounding. High-fold induction was associated with the hypersensitive response (HR), which is triggered only by avirulent stripe rust pathotypes, suggesting that TaAbc1 is a rust-pathotype specific HR-mediator[50]. In our study, we found the Abc1-like family involved in the hypersensitive response against *B*. *zeicola* in maize leaf.

Nudix hydrolases constitute a large family of proteins that hydrolyze nucleoside diphosphate derivatives. Some nudix hydrolases act as ‘housecleaning’ enzymes, whereas others might sense and modulate the levels of their substrates to maintain physiological homeostasis. Expression of the *AtNUDT7* gene is induced by multiple stresses, including biotic and abiotic stresses [51], and AtNUDT7 has been identified as a negative regulator of the defense response; the loss-of-function mutation of AtNUDT7 leads to enhanced disease resistance and causes plants to become hyper-responsive to inciting agents, including pathogenic and nonpathogenic microorganisms [52]. We found that nudix hydrolyse 13 was up-regulated from CP to DP during pathogen infection; thus, we concluded that nudix hydrolyse 13 might act as a negative regulator of the defense response and enhanced disease resistance, thus leading the plants to become hyper-responsive to inciting agents, including pathogens. More interestingly, antifreeze protein was up-regulated during the development of symptoms, as shown by the DGE-seq and qRT-PCR data; thus, it appears that plant antifreeze proteins are homologous to pathogenesis-related proteins and provide protection against psychrophilic pathogens. In addition, the expression and accumulation of AFPs are controlled by developmental regulation and transcriptional factors that are enriched in successive pathogen infection stages.

In summary, using mRNA sequencing and analyzing the differential expression of genes during four disease induction stages, we obtained a genome-wide transcription profile for systemic symptom development in maize leaves infected with *B*. *zeicola*. In addition, the molecular functions of some genes and their associated pathways provide insights into the molecular mechanisms of the maize leaf symptom development in response to *B*. *zeicola* infection. Moreover, the dynamics of transcription factor accumulation during leaf development in response to *B*. *zeicola* infection showed that these transcription factors are likely involved in plant hormone signal transduction. The results obtained will facilitate further investigations of the detailed mechanisms of plant responses to pathogen infection.

## Supporting Information

S1 File The information of primers of differential expression genes (Table A), sequence tags (Table B), differentially expressed transcription factors (Table C) as well as summary of important KEGG pathways (Table D) along the successive development stages influenced by *Bipolaris zeicola* infection. (XLSX)
